# Unsupervised online multitask learning of behavioral sentence embeddings

**DOI:** 10.7717/peerj-cs.200

**Published:** 2019-06-10

**Authors:** Shao-Yen Tseng, Brian Baucom, Panayiotis Georgiou

**Affiliations:** 1 Department of Electrical and Computer Engineering, University of Southern California, Los Angeles, CA, United States of America; 2 Department of Psychology, University of Utah, Salt Lake City, UT, United States of America

**Keywords:** Behavior analysis, Sentence embeddings, Multi-task learning, Unsupervised learning, Couples therapy, Emotion recognition, Emotional embeddings

## Abstract

Appropriate embedding transformation of sentences can aid in downstream tasks such as NLP and emotion and behavior analysis. Such efforts evolved from word vectors which were trained in an unsupervised manner using large-scale corpora. Recent research, however, has shown that sentence embeddings trained using in-domain data or supervised techniques, often through multitask learning, perform better than unsupervised ones. Representations have also been shown to be applicable in multiple tasks, especially when training incorporates multiple information sources. In this work we aspire to combine the simplicity of using abundant unsupervised data with transfer learning by introducing an online multitask objective. We present a multitask paradigm for unsupervised learning of sentence embeddings which simultaneously addresses domain adaption. We show that embeddings generated through this process increase performance in subsequent domain-relevant tasks. We evaluate on the affective tasks of emotion recognition and behavior analysis and compare our results with state-of-the-art general-purpose supervised sentence embeddings. Our unsupervised sentence embeddings outperform the alternative universal embeddings in both identifying behaviors within couples therapy and in emotion recognition.

## Introduction

Representation learning has become a crucial tool for obtaining superior results in many machine learning tasks (Bengio, Courville & Vincent, 2013). In the scope of Natural Language Processing (NLP) notable examples of transforming input into more informative abstractions are *word embeddings* such as *word2vec* (Mikolov et al., 2013) or *GloVe* (Pennington, Socher & Manning, 2014). Word embeddings exploit the use of language by learning semantic regularities based on a context of neighboring words. This form of contextual learning is unsupervised, which allows learning from large-scale corpora and is the main reason for its effectiveness in improved performance on many tasks such as constituency parsing (Tai, Socher & Manning, 2015), sentiment analysis (Dos Santos & Gatti, 2014; Severyn & Moschitti, 2015), natural language inference (Parikh et al., 2016), and video/image captioning (Karpathy & Fei-Fei, 2015; Venugopalan et al., 2016).

With the introduction of sequence-to-sequence models (*seq2seq*) (Sutskever, Vinyals & Le, 2014), embeddings were extended to encode entire sentences and allowed representation of higher level concepts through longer context. For example, Kiros et al. (2015) obtained *sentence embeddings*, which they referred to as *skip-thought* vectors, by training models to generate the surrounding sentences of extracts from contiguous pieces of text from novels. The authors showed that the embeddings were adept at representing the semantic and syntactic properties of sentences through evaluation on various semantic related tasks. Palangi et al. (2016) extracted sentence embeddings from an LSTM-RNN which was trained using user click-through data logged from a web search engine. They then showed that embeddings generated by their models were especially useful for web document retrieval tasks. Later, Tseng, Baucom & Georgiou (2017) extracted sentence embeddings from a conversation model and showed the richness of semantic content by applying an additional weakly-supervised architecture to estimate the behavioral ratings of couples therapy sessions. More recently, Pagliardini, Gupta & Jaggi (2018) learned unsupervised sentence embeddings using an extension of the training objective used in *word2vec* (Mikolov et al., 2013). The authors proposed an unsupervised model which composes sentence embeddings from word vectors and n-gram embeddings through joint optimization. They then showed the generalizability of their sentence embeddings by evaluating on a wide range of downstream NLP tasks.

Sentence representations that are not task-specific but rather *general-purpose* and can be applied directly to multiple NLP tasks have also been proposed. Luong et al. (2016) achieved this by training for various tasks such as machine translation, constituency parsing, and image caption generation, to produce embeddings which improved the translation quality between English and German. Subsequently in Conneau et al. (2017) it was hypothesized that a single Natural Language Inference (NLI) task (MacCartney & Manning, 2014) was sufficient in learning general purpose embeddings due to it being a high-level understanding task. The authors then showed the effectiveness of the sentence embeddings in 12 transfer tasks, examples of which include semantic relatedness, sentiment analysis, and caption-image retrieval. Later, Subramanian et al. (2018) presented a large-scale multitask framework for learning general purpose sentence embeddings by training with a multitude of NLP tasks, including skip-thought training, machine translation, entailment classification, and constituent parsing. Similarly, Cer et al. (2018) proposed a transformer based sentence encoding model trained on multiple tasks which also include skip-thought training, conversational response generation, and NLI.

The benefit of many of the methods in the aforementioned work is that the embedding transformation is learned on large amounts of data. Since the generation of natural language is an extremely complex process, it is crucial to leverage large corpora when training embeddings so as to capture *true* semantic concepts instead of regularities of the data, e.g., domain-specific topics (Klein & Manning, 2005). Previously this was achieved through the use of abundant unlabeled datasets and unsupervised learning techniques (Kiros et al., 2015; Hill, Cho & Korhonen, 2016; Pagliardini, Gupta & Jaggi, 2018). However, as recent work (Subramanian et al., 2018; Cer et al., 2018) has shown, learning sentence representations from multiple labeled datasets can produce significant improvements over prior unsupervised methods.

A common issue with unsupervised training of word or sentence embeddings is the unpredictability of the resulting embedding transformation. In other words, the information carried by embeddings is highly uninterpretable and may often contain redundant or irrelevant information (Jurgovsky, Granitzer & Seifert, 2016). In addition, depending on training conditions such as architecture or dataset, the representations might fail to capture informational concepts or even semantics of the input data (Conneau et al., 2017).

It has also been noted that the quality of sentence embeddings is often highly dependent on the training dataset (Palangi et al., 2016; Tseng, Baucom & Georgiou, 2017). In fact, the benefit of using matched datasets may be so prominent that embeddings trained on small *domain-relevant* datasets could yield results better than those trained on larger generic unlabeled datasets (Kiros et al., 2015). And while many general purpose sentence embeddings have been trained with large amounts of labeled data through multitasking, applications by others to their respective domains might not guarantee the same significant improvement of results. This problem is inherent in the fact that a domain adaptation step is generally still required over the embeddings.

One way that unsupervised representations can better gain domain-specificity is through *multitask learning* (MTL). For example prior work has shown the benefits of leveraging MTL to enhance the informational content of word embeddings in many NLP applications (Collobert & Weston, 2008; Hwang & Sigal, 2014; Bordes, Weston & Usunier, 2014). In recent years, through the advancement of computational methods, MTL has been applied to the learning of sentence embeddings that allow for a larger context window. For example, Yu & Jiang (2016), jointly learned sentence embeddings with an additional pivot prediction task in conjunction with sentiment classification. Rei (2017) predicted neighboring words as a secondary objective to improve accuracy of various sequence labeling tasks.

The *focus of our work is behavior recognition*. We thus target the learning of unsupervised sentence embeddings that are suitable for applications in behavior understanding tasks. Behavior understanding is the complex task of recognizing behavioral cues in human interactions that represent the individuals internal cognitive and psychological state, as well as attitudes, moods, and emotions (Georgiou, Black & Narayanan, 2011; Narayanan & Georgiou, 2013). This requires a high level of natural language understanding and inference in this particular domain, which we hypothesize would be lacking in general purpose embeddings.

Behavior encodes many layers of complexity: the dynamics of the interlocutors, their perception, appraisal, and expression of emotion, their thinking and problem solving intents, skills and creativity, the context and knowledge of interlocutors, and their abilities towards *emotion* regulation (Baumeister et al., 2007). Behavior is not the same as emotions, but it is encoded in part through the modulation of emotional expression and affected by the ability to perceive and regulate emotions, and thus shares a tight relationship with emotional expression. In fact according to some theories (Schacter, Gilbert & Wegner, 2009) emotions are states of feeling that result in physical and psychological changes that influence our behavior. It thus makes sense within our focus task to employ emotion as a task while learning sentence representations.

One of the challenges in MTL is that the labels required by the secondary task are not often available for the vast amounts of unlabeled datasets employed in representation learning. One of the contributions of this work towards this direction is that we do not require the existence of such labeling. Our work differs in that we build on *unsupervised contextual learning* to learn the sentence representation and attempt to guide the sentence embeddings to become domain relevant through a related multitask objective. The underlying assumption of our work is that the behavior expressed in two adjacent sentences will be the same due to short term stationarity. However, the resulting representation encodes a vast amount of information, which we hope to further attune towards domain-relevance. We achieve this through the related task of emotion-related labels. Unlike prior works however, our second emotion-related guiding task does not require prior labeling. We target unsupervised scenarios and use a naive scheme based on limited human-knowledge to *automatically generate multitask labels from unlabeled data in an online manner*. We hypothesize that by adopting an extremely simple form of sentiment analysis (Pang & Lee, 2008) as the multitask objective the unsupervised sentence embeddings will become more adept in behavior understanding.

Specifically in this work we aspire to combine the advantages of unsupervised learning with multitask learning to derive representations that are better suited for affect and behavior recognition tasks. We propose an online MTL framework which aims to *guide* unsupervised sentence embeddings into a space that is more discriminative in the targeted application scenario even under the use of mismatched and limited data. In our framework, transfer of domain-knowledge is achieved through an additional task in parallel with contextual learning. The labels for the multitask are generated online from the data to maintain an unsupervised scenario. We show that embeddings trained through this framework offer improved deftness in multiple supervised affective tasks.

## Unsupervised Multitask Embeddings

In this section we describe the methods used to learn domain-adapted unsupervised sentence embeddings. We introduce the learning of sentence embeddings using sequence-to-sequence models followed by the formulation of our online multitask training objective and its architecture.

### Sequence-to-sequence sentence embeddings

The sequence-to-sequence model maps input sequences to output sequences using an encoder–decoder architecture. Given an input sentence **x** = (*x*_0_, *x*_2_, …, *x*_*T*_) and output sentence **y** = (*y*_0_, *y*_2_, …, *y*_*T*′_), where *x*_*t*_ and *y*_*t*_ represent individual words, the standard sequence model can be expressed as computing the conditional probability

(1) \begin{eqnarray*}P(\mathbf{y}{|}\mathbf{x})=\prod _{t=0}^{{T}^{{^{\prime}}}}P({y}_{t}{|}{y}_{i\lt t},\mathbf{s},h)\end{eqnarray*}

where **s** is the sequence of outputs *s*_*t*_ from the encoder and *h* is the internal representation of the input given by the last hidden state of the encoder. For a given dataset $\mathcal{D}={\{({\mathbf{x}}_{n},{\mathbf{y}}_{n})\}}_{n=1}^{N}$, we denote the learned internal representation as

\begin{eqnarray*}{h}_{\theta }\equiv f(\mathbf{x}{|}\mathcal{D})=f(\mathbf{x}{|}\theta ) \end{eqnarray*}

where *f*(⋅) is the encoder function and *θ* is the set of parameters resulting from $\mathcal{D}$. The internal representation *h*_*θ*_ encodes the input **x** into a vector space that allows the decoder to generate a good estimate of **y**. In cases where $\mathcal{D}$ contains semantically-related data pairs, *h*_*θ*_ can be viewed as a semantic vector representation of the input, or sentence embedding, which can be useful for subsequent NLP tasks. In our case we apply contextual learning and designate consecutive sentences in continuous corpora as **x** and **y**.

While this model allows us to obtain semantically rich embeddings through training on unlabeled data, the quality of the embeddings is highly influenced by biases in the data and prevents the embeddings from becoming specialized in any target task (Conneau et al., 2017). Therefore we propose to enhance the quality of unsupervised sentence embeddings through multitask learning.

### Multitask embedding training

The addition of a multitask objective can guide embeddings into a space that is more discriminative in a target application. We hypothesize that this holds true even when the multitask labels are generated online from unlabeled data with no assumption of label reliability, as long as there is some relation between the multitask and target application.

**Figure 1:**
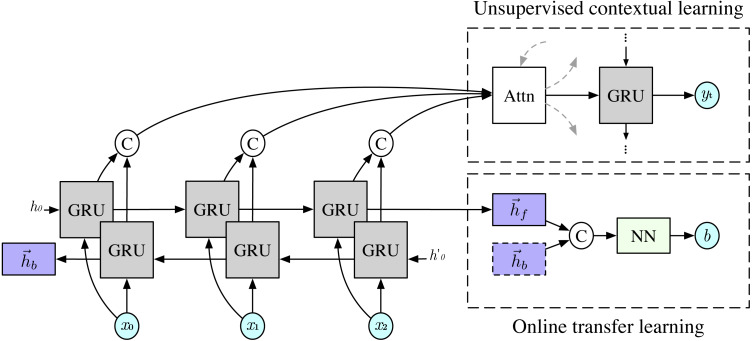
Bidirectional sequence-to-sequence conversation model with multitask objective. The GRU blocks represent multi-layered RNNs using GRU units, C is the concatenation function, and Attn is an attention mechanism Bahdanau, Cho & Bengio (2014) with dotted arrows representing connections to and from other timesteps. For simplicity, only one timestep (*y*_*t*_) of the decoder is shown.

Assuming an online system which generates multitask labels **b** for each input **x** we can augment the dataset to yield ${\mathcal{D}}_{\mathrm{aug}}={\{({\mathbf{x}}_{n},{\mathbf{y}}_{n},{\mathbf{b}}_{n})\}}_{n=1}^{N}$. We then aim to predict this new label **b** in conjunction with the original output sequence **y**. This is implemented in our *seq2seq* model by adding another *head* to the internal representation *h*, shown in Fig. 1, which we will refer to as the *multitask network*. In addition to Eq. (1), the model now also estimates the conditional probability

\begin{eqnarray*}P(\mathbf{b}{|}\mathbf{x})=g(h{|}{\mathcal{D}}_{\mathrm{aug}})=g({{h}_{\theta }}_{\mathrm{aug}}) \end{eqnarray*}

where *g*(⋅) is the network function for online transfer learning using the multitask network and *h*_*θ*_aug__ is the new internal representation given by ${\mathcal{D}}_{\mathrm{aug}}$. In this work, *g*(⋅) is implemented using a multilayer perceptron. The overall architecture is shown in Fig. 1.

The training loss is then the weighted sum of losses from the multiple tasks, defined as

\begin{eqnarray*}J=\lambda \cdot {L}_{1}(\mathbf{y},\mathbf{x})+(1-\lambda )\cdot {L}_{2}(\mathbf{b},\mathbf{x}) \end{eqnarray*}

where *L*_1_ and *L*_2_ are the cross entropy losses for contextual learning and the additional task, respectively. With most multitask setups there is an issue on how to control the training ratio *λ* to account for different data sources. For example, if there is no overlap in inputs of the multiple tasks then *λ* can only alternate between 0 and 1 during training to switch between the different tasks. However, since we propose a multitask objective whose labels are generated from incoming data we are able to freely adjust *λ*. It is possible to adjust the multitask ratio as training progresses to put emphasis on different tasks but we do not make any assumptions on the optimal weighting scheme and give equal importance to both tasks by setting *λ* to 0.5.

### Online multitask label generation

To guide the embeddings in becoming more suitable for affective tasks, we select a multitask objective that classifies the polarity in sentiment (positive or negative) of input sentences. Tasks such as emotion recognition or human behavior analysis (Narayanan & Georgiou, 2013) are more complicated than these two affective states, however we hypothesize this is a related task allowing for domain knowledge transfer into the sentence embeddings.

We generate the affective labels for each input during training using an online mechanism. In our online approach we apply the simplest method by automatically labeling inputs using a simple, knowledge-driven, look-up table of likely affect of single words (Tausczik & Pennebaker, 2010). Specifically, we use words categorized in the two top-level affective states: negative and positive emotion. An input sentence is assigned a *Negative* or *Positive* label based on the majority number of words corresponding to each affective state. Some examples of affective words in the affective look-up table are shown in Table 1.

**Table 1:** Examples of positive and negative affect words.

Affective state			
Positive		Negative	
cute	love	ugly	hate
rich	nice	hurt	nasty
special	sweet	wicked	distraught
forgive	handsome	shame	overwhelm

Evidently, this labeling approach differs slightly from sentiment analysis (Pang & Lee, 2008), which mostly focuses on classifying the polarity of subjective opinions. In our case we label all the inputs naively based on the count of affective words and do not consider semantic context or even simple word negation. We expect this approach to deviate greatly from the ground truth, and that truth may be contextual, subjective, and fluid, however we hypothesize the inclusion of affective knowledge in embeddings will still be beneficial in identifying more complex behaviors or emotions later. Specifically, we do not want to constrain the system through methods such as (Pang & Lee, 2008) but rather place emphasis and focus on domain relevant terms.

## Evaluating on behavior identification using embeddings

After MTL training, the encoder in the *seq2seq* model is used to extract embeddings for use as features in behavior identification in long pieces of text (which we refer to as sessions). Each session has a behavior label and contains multiple sentence embeddings. We define sentence embeddings to be the concatenation of the final output states of both the forward and backward RNNs in the encoder. We also concatenated the output states from all the intermediate layers of the encoder. This is an extension of history-of-word embeddings (Huang et al., 2018) and is motivated by the intuition that intermediate layers represent different levels of concept. By utilizing intermediate representations of the sentence, we expect that more information related to human behavior can be captured.

To evaluate the ability of the proposed system in creating behavior-tuned embeddings we apply the embeddings to task of behavior and emotion analysis. We do this in multiple ways: from minimal information about the domain, to training supervised neural networks over the unsupervised sentence embeddings. These methods are described below.

### Unsupervised clustering of embeddings

As an initial evaluation step we analyzed the performance of the embeddings on a binary behavior classification task using minimal training on the Couples Therapy Corpus which will be described below. We applied a simple k-means clustering method on sentence embeddings from training sessions to obtain two clusters. We then labeled the clusters by randomly selecting a single session from the training set as seed and assigning the session label to the cluster which the majority of embeddings in that session belonged to. The other cluster was subsequently labeled as the opposite class label. Final test session labels were predicted based on which cluster the majority of embeddings from a session were in. Although this method of behavior classification is very rudimentary with the possibility the randomly selected session being an outlier, it nonetheless gives valuable insight on the discriminative power of the sentence embeddings. It should be noted that we do not make any assumptions on the meaning behind the clusters in this work other than their adeptness in classifying behavior.

### Embeddings as features in supervised learning

We also evaluated two supervised techniques on both the IEMOCAP and Couples Therapy Corpus. The two methods are k-nearest neighbor and a more advanced neural network-based method, both of which utilize the unsupervised embeddings as features in supervised learning.

#### k-Nearest neighbors

In this evaluation scenario we used the labels in the training data towards constructing a very simple classifier using the k-nearest neighbors (k-NN). All embeddings in the training set were assigned the label of the session they belonged to. A test embedding was then labeled according to its k-nearest neighbors in the training set. The final session label was obtained by a majority vote over all embeddings in the session.

#### Neural networks

Finally, we employed neural networks to estimate behavior ratings as well as recognize emotions. For behavior annotation we applied the framework proposed in Tseng, Baucom & Georgiou (2017). Sessions were segmented into sentences and represented as a sequence of embeddings. A sliding window of size 3 was applied over the embeddings followed by an RNN using LSTM units. LSTM units were used instead of GRUs, which were used in the *seq2seq* model, to allow direct comparison with results from Tseng, Baucom & Georgiou (2017). However we do not expect significant differences in performance between the two types of units, as was shown by Chung et al. (2014) in their own applications.

The network was trained to predict the session rating from each window of multiple sentences representations. The final rating was obtained by training a Support Vector Regressor to map from the median value of all window predictions in a session to the session rating.

## Experimental Setup

### Datasets

In this section we describe the datasets that were used in the experiments. We used the OpenSubtitles2016 corpus (Lison & Tiedemann, 2016) to pre-train sentence embeddings in the online multitask framework. To evaluate the embeddings in domain-specific tasks, we used the Couples Therapy Corpus (Christensen et al., 2004) and IEMOCAP (Busso et al., 2008)^1^. 1The dataset OpenSubtitles2016 can be downloaded from http://opus.nlpl.eu/OpenSubtitles-v2018.php. IEMOCAP can be obtained from SAIL USC by visiting https://sail.usc.edu/iemocap/iemocap_release.htm. The Couples Therapy Corpus involves human subjects participating in real couple therapy interactions and as such is protected under an Institutional Review Board (IRB). Information on obtaining IRB clearance and access to the corpus can be obtained by contacting the authors.

#### OpenSubtitles

Since our final task is emotion and behavior analysis of human interactions, we applied a dataset that contains conversational speech to pre-train our embeddings. A natural choice for a source rich in dialogue is subtitles from movies and TV shows. To this end we used the OpenSubtitles2016 corpus (Lison & Tiedemann, 2016) to train the unsupervised sentence embeddings.

The OpenSubtitles2016 corpus was compiled from a database dump of the opensubtitles.org repository and comprises of subtitles from 152,939 movies and TV episodes spanning a time period of over 20 years. Out of more than 60 languages in the corpus we selected only subtitles in the English language for use in our training. The original corpus applied basic pre-processing through text standardization and segmentation of the subtitles into sentences (Tiedemann, 2009). We then used further techniques to clean up the text by applying auto-correction of commonly misspelled words, contraction removal, and replacement of proper nouns through parts-of-speech tagging.

To generate back-and-forth conversations we assigned consecutive sentences in the subtitles as turns in an interaction. Since there is no speaker information in the corpus, distinguishing between dialogues and monologues without the use of more advanced content analysis methods is nontrivial. However, we assume that this difference in conversational continuity will be dampened by the large amount of data available. We also reason that monologues also represent some form of internal dialogue which also ties the concepts between sentences. More importantly, since our final task is to represent behavior, we desire that sentence pairs carry information related to behavior. This can be achieved through the concept of *short-term behavior stationarity* in which two nearby sentences are likely to represent the same behavior, irrespective of turn-taking. This property was also shown by Black et al. (2013) wherein correlations in behavior were observed across interlocutors.

After forming all utterance/reply pairs from the corpus we randomly sampled 30 million sentence pairs as the final training data.

#### Couples therapy corpus

We evaluated our sentence embeddings in the task of annotating behaviors in human interactions using data from the UCLA/UW Couple Therapy Research Project (Christensen et al., 2004). This corpus pertains to the training of unsupervised, k-NN, and neural network learning methods described in the previous section.

The Couples Therapy Corpus contains recordings of 134 real couples with marital issues interacting over multiple sessions. In each session the couples each discussed a self-selected topic for around 10 min. The recordings of the session were then rated by multiple annotators based on the Couples Interaction (Heavey, Gill & Christensen, 2002) and Social Support (Jones & Christensen, 1998) rating systems. The combined rating system describes 31 behavioral codes rated on a Likert scale of 1 to 9, where 1 indicates strong absence and 9 indicates strong presence of the given behavior. The number of annotators per session ranged from 2 to 12, however the majority of sessions (∼90%) had 3 to 4 annotators. Annotator ratings were then averaged to obtain a 31 dimensional vector of behavior ratings per interlocutor for every session. The ratings were binarized to produce labels for the classification task and the Likert scale values were used for behavior rating estimation.

In this work we focused on the behaviors *Acceptance*, *Blame*, *Humor*, *Sadness*, *Negativity*, and *Positivity*. While the behaviors *Negativity* and *Positivity* are more certain to benefit from the affect labels in MTL, which may be loosely similar, the remaining behaviors have more specific definitions which may be more challenging in identifying. We formulated two tasks for each of the behaviors: (1) binary classification on the presence of a behavior and (2) regression on the rating of a behavior in the whole session.

Similar to prior works (Chakravarthula et al., 2015; Tseng, Baucom & Georgiou, 2017) we used only those sessions that had averaged ratings in the top and bottom 20% of the dataset. In total, 85 individual couples were included in our evaluation dataset. Evaluation of the models was performed using a leave-one-*couple*-out cross-validation scheme. That is, for each fold, sessions from one couple were used as the test set while the remaining sessions were used as the training and validation set. We report evaluation metrics averaged across these 85 folds.

#### IEMOCAP

We also evaluated the effectiveness of our sentence embeddings in emotion recognition using the Interactive Emotional Dyadic Motion Capture Database (IEMOCAP) (Busso et al., 2008). We use this corpus for domain-supervised learning using the embeddings as features. This dataset contains recordings from five male–female pairs of actors performing both scripted and improvised dyadic interactions. Utterances from the interactions were then rated by multiple annotators for dimensional and categorical emotions. Similar to other works (Fayek, Lech & Cavedon, 2017; Cho et al., 2018), we focused on four categorical labels where there was majority agreement between annotators: *happiness*, *sadness*, *anger*, and *neutral*, with *excitement* considered as *happiness*. We used the transcripts from the dataset and removed any acoustic annotations such as “laughter” or “breathing”. After discarding empty sentences our final dataset consisted of 5,500 utterances (1,103 for *anger*, 1,078 for *sadness*, 1,615 for *happiness*, and 1,704 for *neutral*). To evaluate the domain-supervised layers we used a leave-one-*pair*-out cross-validation testing scheme and report the evaluation metrics averaged across 5 folds.

### Model architectures and training details

#### Sentence embeddings

The sequence-to-sequence model with multitask objective comprises three sections: encoder, decoder, and the multitask network. The encoder was implemented using a multi-layered bidirectional RNN using GRU units. We performed a grid search using hyper-parameter settings of two and three layers, and 100 and 300 dimensions in each direction per layer. For the decoder a unidirectional RNN using GRU units was used instead of bidirectional. The number of layers in the decoder were the same as the encoder while the dimension size was doubled to account for the concatenation of states and outputs from both directions.

The multitask network was implemented using a neural network with four hidden layers of sizes 512, 512, 256, and 128. The final output had a dimension size of 2 to represent *Positive* and *Negative* affect class labels. We used the rectified linear unit (ReLU) function as activation functions in the hidden layers and a softmax activation function in the final output layer. No other network hyper-parameters were tried for the multitask network.

The sentence embedding models were trained with the OpenSubtitles dataset for five epochs using stochastic gradient descent with an added momentum term. The learning rate was set to 0.05 and momentum set to 0.9. We also reduced the learning rate by a factor of 10 every epoch.

#### Supervised behavior annotation

Similar to Tseng, Baucom & Georgiou (2017) we used a recurrent neural network to estimate behavior ratings in the Couples Therapy Corpus. The network had a single recurrent layer implemented using LSTM units with dimension size 50. A sigmoid function was applied before the output to estimate the normalized rating value. In each fold one couple was randomly selected as validation to select the best model.

#### Supervised emotion recognition

A neural network with four hidden layers was used to classify emotions using embeddings of sentences from the IEMOCAP dataset. The hidden layers were of size 256 and used ReLU as the activation function. The model was trained for 20 epochs using Adagrad (Duchi, Hazan & Singer, 2011) as the optimization method. No other network hyper-parameters were tried for the emotion recognition network. A subset of the training data (∼10%) was used as validation in selecting the best model.

## Experimental Results

We evaluated the performance of our unsupervised multitask sentences embeddings on the task of behavior annotation in the Couples Therapy Corpus, as well as emotion recognition on the IEMOCAP dataset. We also compared to multiple state-of-the-art general purpose embeddings such as InferSent (Conneau et al., 2017), GenSen (Subramanian et al., 2018), and Universal Sentence Encoder (Cer et al., 2018).

### Results on couples therapy corpus

We defined two sub-tasks in behavior annotation on the Couples Therapy Corpus: (1) binary classification of the presences of behaviors and (2) regression for real-valued session ratings of the behaviors.

For the classification sub-task we used the accuracy averaged across all test folds as the evaluation metric. Table 2 shows the accuracy results on different behaviors in the Couples Therapy Corpus. The addition of the multitask objective improved the classification accuracy of sentence embeddings from the conversation model across all behaviors except *Positivity* in unsupervised classification with k-Means. Under supervised learning using k-NN, our multitask embeddings improved accuracy on all behaviors except *Humor*. In terms of mean accuracy, our multitask embeddings performed better than other sentence embeddings with an absolute improvement over no multitasking of 1.07% and 3.24% for unsupervised and supervised methods respectively. Our multitask embeddings also achieved the highest mean accuracy over all the behaviors. The improvement over the second best results obtained from GenSen was statistically significant with *p*-value <0.006 using McNemar’s test.

**Table 2:** Accuracy (%) of behavior identification using sentence embeddings. The improvement of our model over the next best performing model across all behaviors is statistically significant with *p* < 0.006.

Method	Embedding model	Acceptance	Blame	Humor	Negativity	Positivity	Sadness	Mean accuracy
k-Means	InferSent (Conneau et al., 2017)	58.9	63.6	60.7	61.4	62.1	58.9	60.93
	GenSen (Subramanian et al., 2018)	53.9	66.4	58.9	61.4	61.4	59.6	60.27
	Universal Sentence Encoder (Cer et al., 2018)	59.3	65.7	59.6	61.8	64.3	59.6	61.72
	Conversation Model (Tseng, Baucom & Georgiou, 2017)	61.9	65.4	59.1	64.6	**65.7**	57.9	62.43
	+ Online MTL (proposed)	**64.0**	**66.4**	**62.1**	**65.0**	62.1	**61.4**	**63.50**
k-NN	InferSent (Conneau et al., 2017)	83.2	81.1	57.1	85.4	78.6	65.7	75.27
	GenSen (Subramanian et al., 2018)	**85.0**	85.0	56.1	85.7	81.1	63.2	76.02
	Universal Sentence Encoder (Cer et al., 2018)	80.0	82.5	**60.4**	83.9	79.6	66.8	75.53
	Conversation Model (Tseng, Baucom & Georgiou, 2017)	79.6	80.0	59.6	85.7	82.5	64.6	75.53
	+ Online MTL (proposed)	**85.0**	**85.4**	60.0	**87.9**	**86.8**	**67.9**	**78.77**

For the regression sub-task we evaluated performance using Krippendorff’s alpha coefficient (Hayes & Krippendorff, 2007). Krippendorff’s alpha is a reliability measure of the agreement between independent observers in regards to their annotation of data, commonly known as the inter-annotator agreement. We used this metric to evaluate how well trained models would function as a replacement for human annotators. Similar to Tseng, Baucom & Georgiou (2017) we evaluate the agreement with various ways of incorporating machine-generated ratings. In the first method, human annotations were randomly replaced by the estimated ratings in each session. This was performed 10 times to obtain the average Krippendorff’s alpha of random injection. In the second method, the outlier annotation (rating farthest from the mean) in each session was replaced by the estimated ratings.

Table 3 shows the inter-annotator agreement of the different injection methods. While no system was consistently optimal, we observed that our online MTL embeddings were comparable with state-of-the-art general purpose embeddings. In fact, statistical tests using Mann–Whitney *U* test on the annotation errors showed no significant differences between the best model and ours.

**Table 3:** Inter-annotator agreement (Krippendorff’s alpha) of estimated behavior ratings using different incorporation methods. There is no statistical significance in differences between models, however all models have significant improvement over randomly generated ratings.

Method	Model	Acceptance	Blame	Humor	Negativity	Positivity	Sadness
	Human	0.790	0.828	0.584	0.829	0.695	0.623
Random injection	Random ratings	0.387	0.443	0.161	0.522	0.384	0.274
	InferSent (Conneau et al., 2017)	**0.790**	**0.828**	0.455	**0.829**	**0.695**	0.455
	Gensen (Subramanian et al., 2018)	0.736	0.773	0.452	0.772	0.649	0.460
	Universal Sentence Encoder (Cer et al., 2018)	0.742	0.773	**0.457**	0.778	0.643	**0.472**
	Conversation model (Tseng, Baucom & Georgiou, 2017)	0.722	0.757	0.442	0.782	0.644	0.462
	+ Online MTL (proposed)	0.735	0.773	0.450	0.787	0.645	0.468
Worst-annotation-out	Random ratings	0.341	0.405	0.127	0.521	0.392	0.304
	InferSent (Conneau et al., 2017)	0.790	**0.829**	**0.584**	0.829	0.695	0.563
	Gensen (Subramanian et al., 2018)	0.804	0.820	0.565	0.844	**0.731**	0.559
	Universal Sentence Encoder (Cer et al., 2018)	**0.814**	0.818	0.575	0.846	0.726	0.574
	Conversation model (Tseng, Baucom & Georgiou, 2017)	0.786	0.796	0.568	0.856	0.725	0.572
	+ Online MTL (proposed)	0.801	0.815	0.567	**0.861**	0.727	**0.578**

**Figure 2:**
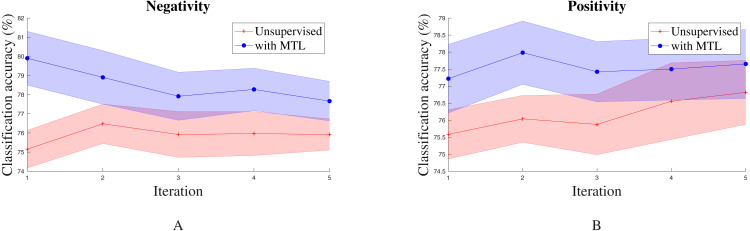
Standard error plot of classification accuracy on *Negativity* and *Positivity* for various model hyper-parameter configurations across multiple iterations. Other than *Humor* and *Sadness*, other behaviors exhibit similar trends.

To factor out the influence of hyper-parameters and randomness of training we analyzed the performance of all the *seq2seq* models in our hyper-parameter search space. For each model configuration, five intermediate checkpoints from training were randomly selected. Sentence embeddings were then extracted from these individual models and applied to the behavior classification task. We then compared the performance of models with and without multitask learning. The standard error plot of the performance in *Positivity* and *Negativity* recognition is shown in Fig. 2. We observed that the addition of the multitask learning objective collectively increased performance in the final task for most behaviors. This shows that the addition of online transfer learning through multitask to unsupervised sentence embeddings does indeed provide an advantage in performance.

### Results on IEMOCAP

We evaluated the performance of emotion recognition on IEMOCAP using weighted accuracy (WA) which avoids inflation due to imbalanced number of labels in each class. This is also equivalent to the macro-average of recall scores per class. In addition to general purpose embeddings we also compared with other works that only used IEMOCAP transcripts (Cho et al., 2018; Jin et al., 2015; Gamage, Sethu & Ambikairajah, 2017). It should be noted that there is no official consensus on train/test split or evaluation procedure in IEMOCAP, and while we made every effort to be consistent with past work (in terms of label classes, number of utterances used, and cross-validation scheme) the results may not be exactly comparable.

The results of emotion recognition on IEMOCAP are shown in Table 4. We observed that the addition of online MTL improved the accuracy of conversation model embeddings by an absolute value of 8.02%, which is more than 14% relative improvement. When comparing among our own implementations we observed that the highest accuracy was obtained using embeddings from the Universal Sentence Encoder which had a weighted accuracy of 64.83%. The system trained using our sentence embeddings offered a close second by less than one percent with 63.84% accuracy. Statistical analysis using McNemar’s test showed that the improvement of the best system over our proposed embeddings was not significant. However, we observed significant improvement from our model over embeddings from InferSent with *p*-value <0.02. Given the considerably smaller amount of pre-training data required and the simpler structure of our proposed MTL system this similarity in performance to Universal Sentence Encoder and advantage over other embeddings is notable.

**Table 4:** Weighted accuracy of emotion recognition on IEMOCAP.

Method	WA (%)
Lex-eVector (Jin et al., 2015)	57.40
E-vector + MCNN (Cho et al., 2018)	59.63
mLRF (Gamage, Sethu & Ambikairajah, 2017)	63.80
InferSent (Conneau et al., 2017) + DNN	62.60
GenSen (Subramanian et al., 2018) + DNN	60.62
Universal Sentence Encoder (Cer et al., 2018) + DNN	**64.83**
Conversation Model (Tseng, Baucom & Georgiou, 2017) + DNN	55.82
+ Online MTL (proposed) + DNN	63.84

## Conclusion

In this work we explored the benefits of introducing additional objectives to unsupervised contextual learning of sentence embeddings. We found empirical evidence that supports the hypothesis that MTL can increase the affective deftness of unsupervised sentence embeddings, even when the multitask labels are generated online using a naive knowledge-driven approach.

Our proposed model has the benefit of not requiring additional effort in generating or collecting data for multitask training. This allows learning from large-scale corpora in an unsupervised manner while simultaneously applying transfer learning. In contrast to general purpose sentence embeddings, our model for learning sentence representations is less complex and requires less training effort, while at the same time yields similar or higher performance in our target task. Through this work we have shown that there are benefits in adopting guided unsupervised learning during embedding pre-training instead of overemphasis on universal applications.

While we do expect that further improvements can be obtained through better labels for the multitask objective, that would entail additional effort in system design and label generation while not undermining our conclusions. In addition, we also expect that multitask labels that are too domain-specific (e.g., focusing on a specific way or definition of affective expression) may actually hinder the performance of unsupervised embeddings. We will expand on this direction through additional tasks in our multitask framework in future work.

## Additional Information and Declarations

### Data Availability

The following information was supplied regarding data availability: The code used in this work is provided at
https://bitbucket.org/georgiou/unsupervised_mt_behavioral_sentence
The dataset OpenSubtitles2016 can be downloaded from http://opus.nlpl.eu/OpenSubtitles-v2018.php. IEMOCAP can be obtained from SAIL USC by visiting https://sail.usc.edu/iemocap/iemocap_release.htm. The Couples Therapy Corpus involves human subjects participating in real couple therapy interactions and as such is protected under an Institutional Review Board (IRB). Information on obtaining IRB clearance and access to the corpus can be obtained by contacting the authors.
